# Food Taboos and Cultural Beliefs Influence Food Choice and Dietary Preferences among Pregnant Women in the Eastern Cape, South Africa

**DOI:** 10.3390/nu11112668

**Published:** 2019-11-05

**Authors:** Gamuchirai Chakona, Charlie Shackleton

**Affiliations:** Department of Environmental Science, Rhodes University, Grahamstown 6140, South Africa; c.shackleton@ru.ac.za

**Keywords:** pregnancy, food taboos, cultural beliefs, isiXhosa, food practices, dietary diversity, maternal and child nutrition

## Abstract

A well-nourished and healthy population is a central tenet of sustainable development. In South Africa, cultural beliefs and food taboos followed by some pregnant women influence their food consumption, which impacts the health of mothers and children during pregnancy and immediately afterwards. We documented food taboos and beliefs amongst pregnant isiXhosa women from five communities in the Kat River Valley, South Africa. A mixed-methods approach was used, which was comprised of questionnaire interviews with 224 women and nine focus group discussions with 94 participants. Overall, 37% of the women reported one or more food practices shaped by local cultural taboos or beliefs. The most commonly avoided foods were meat products, fish, potatoes, fruits, beans, eggs, butternut and pumpkin, which are rich in essential micronutrients, protein and carbohydrates. Most foods were avoided for reasons associated with pregnancy outcome, labour and to avoid an undesirable body form for the baby. Some pregnant women consumed herbal decoctions for strengthening pregnancy, facilitating labour and overall health of both themselves and the foetus. Most learnt of the taboos and practices from their own mother or grandmother, but there was also knowledge transmission in social groups. Some pregnant women in the study may be considered nutritionally vulnerable due to the likelihood of decreased intake of nutrient-rich foods resulting from cultural beliefs and food taboos against some nutritious foods. Encouraging such women to adopt a healthy diet with more protein-rich foods, vegetables and fruits would significantly improve maternal nutrition and children’s nutrition. Adhering to culturally appropriate nutrition education may be an important care practice for many pregnant women in the Kat River Valley.

## 1. Introduction

Nutritional status is considered a key indicator of national development because a well-nourished and healthy population is considered a moral obligation, it concords with human rights and it is a precondition for sustainable social, economic and human development. Poor health due to limited nutrition affects individual well-being and human dignity. In sub-Saharan Africa, food insecurity and malnutrition remain a major challenge [1], with women of reproductive age (particularly pregnant women) and children under the age of five being at high risk [2,3,4,5]. Many women in sub-Saharan Africa remain particularly exposed to what has become known as “hidden hunger.” “Hidden hunger” is the lack of, or inadequate intake of micronutrients, resulting in different types of malnutrition, such as anaemia and deficiencies of iron, vitamin A and zinc [1,6,7], among others. This can occur even in the presence of adequate energy and protein intake. Globally, about 9.8 million women are vitamin A deficient [8], and iron-deficiency anaemia contributes to at least 18% of maternal deaths in developing countries [8,9]. Food insecurity in the form of undernutrition also affects early childhood development, as the child’s brain and central nervous system are compromised [10,11]. This can lead to poor school performance, reduced physical work capacity and decreased productivity later in life. The first 1000 days of a child’s life are critical for optimal growth, health and development; therefore, intake of diverse and good quality food, in sufficient quantity, is crucial [8]. 

In some societies within sub-Saharan Africa, women and young children are prohibited from eating certain foods due to ethnic or cultural beliefs and taboos, and those may include micronutrient rich foods [12,13,14,15,16,17,18,19]. Food taboos are known from almost all human societies as a systematised set of rules about which foods or combinations of foods one may not consume [20]. However, food taboos often target pregnant women to prevent what is perceived as harmful effects of these foods on the new-born [12,13]. For example, in Ethiopia [15,16,17], Gambia [12], Nigeria [13,18] and in Gabon and the Democratic Republic of Congo [19], pregnant women are usually forbidden from consuming the richest food-sources of iron, carbohydrates, animal proteins and micronutrients. This is mainly because of the fear that the child may develop bad habits after birth or may be born with diseases, the fear of delayed labour due to large babies and the beliefs that certain foods stimulate continuous menstruation, leading to infertility in women. Food taboos have also been reported in low and middle-income countries in Asia [21,22]. These strong taboos may limit the quantities and quality of food a pregnant woman may choose to consume. For example, protein-rich foods in the form of meat, fish, eggs and legumes are often denied to pregnant women in various parts of Africa, and in many other populations [14,19,23,24,25,26]. However, studies regarding that are mostly within an ethnobotany or anthropology framework, and thus, have rarely investigated the nutritional limitations of such practices.

Although different societies have traditional beliefs regarding harmful foods for women during pregnancy, they also have foods that are regarded as beneficial for a range of reasons. In some African societies, pregnant women, lactating mothers and children are encouraged to diversify their diets through the use of wild foods to promote maternal and child health and improve child development [27,28,29]. For example, Nigerian pregnant women consume zinc-rich seeds in their porridge, and the leaves and the barks of different trees, which are good sources of vitamins, calcium, copper, iron, zinc, some protein and fat, to increase breastmilk production, expel intestinal worms and to increase weight-gain in infants [27]. Some wild plants are consumed to ease delivery, for breastmilk stimulation, for prevention of anaemia and for the “strengthening” of the foetus; thus, improving overall health for both the mother and foetus [28,29]. 

Promoting maternal and child health and nutrition is an important public health concern, especially in low-income countries. An adequate supply of nutrients at all stages of pregnancy is important for the maintenance of a delicate balance between the needs of the mother and those of the foetus for optimal development and function in both [30]. Little is known about food restrictions and the use of wild foods during pregnancy in South Africa. 

South Africa is regarded as a food-secure nation, but large numbers of households within the country are food insecure; there are high rates of malnutrition in both children and adult women [4,5]. With more than half of the population living in poverty and a third in extreme poverty [31], it is difficult for many South African households to purchase enough good-quality food to feed the entire household. However, if food is available in the household, cultural determinants of what is being consumed by pregnant women may prohibit them from the nutritious foods available. Therefore, the present study focused on pregnant women and mothers in the Kat River Valley in South Africa, documenting the taboo foods that are culturally prohibited from being eaten during pregnancy and the reasons for that. The study also sought to understand if there were any specific foods that pregnant women were encouraged to consume for maternal health and to promote their children’s growth and development. By generating knowledge on dietary practices and food taboos by pregnant women, the study determined whether they have any potential for adverse effects or nutritional benefits on the growth and development of the unborn child. This will help in establishing cultural competency in the nutrition programmes that will involve the delivery of maternal and child health care services which might be applied in South Africa and globally. 

## 2. Materials and Methods 

### 2.1. Study Area

The study was conducted in five settlements (Hertzog (32°34′38.07″ S, 26°42′50.88″ E), Balfour (32°32′32.13″ S, 26°40′23.06″ E), Ekuphumuleni (32°32′50.78″ S, 26°39′13.83″ E), Blinkwater (32°41′57.36″ S, 26°35′14.42″ E) and Ntilini (32°41′25.53″ S, 26°36′21.42″ E)) along the Kat River Valley in the Eastern Cape province of South Africa. The Kat River Valley is bounded by the Klein Winterberg mountain range to the west, the Katberg and Cathcart mountains to the north-east and the Hogsback range to the east, and to the south-west is Fort Beaufort, a small farming town at the foothills of the Winterberg and the Amatole Mountains. The Kat River catchment is about 80 km long and covers an area of about 1715 km^2^. The area is home to about 50,000 people, mainly IsiXhosa [32], which made it ideal for a study involving cultural beliefs and indigenous knowledge. The majority of the households are female-headed. Livestock ownership is one of the most important assets and livelihood activities in Kat River Valley, with high numbers of sheep and goats and few cattle per household [33]. However, the majority of households do not practise crop production and rely on food purchasing. Education levels are poor, with approximately only 9.7% of the population having a matric qualification (matric qualification refers to the final year of high school and the qualification received on graduating from high school which gives a person the minimum university entrance requirement) and a countable number having a tertiary qualification [32]. The area is characterized by extremely low formal employment rates [32]. Government social grants are the most common sources of cash income, with 90% of households receiving one or more old age pensions of R1570 per month or child support grants of R480 per month. The South African Rand to US Dollar exchange rate was approximately 12:1 at the time of data collection.

### 2.2. Sampling

This study was conducted in February and March 2018. A ‘mixed suite’ [34] of research tools, including both quantitative and qualitative techniques, was used. These included household surveys to collect quantitative data needed for the study through questionnaires and focus group discussions, where qualitative data was also collected. Focus group discussions complemented household surveys. These added more insights into collective meanings attached to food-consumption practices by pregnant women in the Kat River Valley that could not be elicited by questioning individuals. With these group interactions, we developed an understanding of the meaning and experiences of peoples’ lives from the point of view of those who experience it. All interviews were conducted in the respondent’s preferred language of isiXhosa or English. Both translators and enumerators were trained on how to conduct interviews using the questionnaire, so as to provide full understanding of the questions administered. Ethics approval was granted by the Rhodes University Ethical Standards Committee on 6 November 2017 with reference number 8628531. The study’s subjects provided written or oral consent before each interview and each focus group discussion.

#### 2.2.1. Household Surveys

Women’s dietary data were obtained through the administration of questionnaires to selected households within the Kat River Valley. To calculate the sample size, we determined the population size of our study area, which was 50,000 inhabitants, and we specified a margin of error of 5% (ᾳ = 0.05) and 90% confidence level (β = 0.10 for power), where we assumed that the actual population mean falls within the confidence interval. We then used a standard deviation of 0.5, which showed the expected variance of responses. In our case, the sample size calculated was *n* = 268 and the authors subsequently rounded this to 280 women. We calculated the sample size to have an idea of the number of participants that would be ideal for the study. However, a purposive sampling approach [35] was used with a target to reach out to 280 women. The researcher decided on what needed to be known and set out to find people who could and were willing to provide the information by virtue of their knowledge or experience [35]. In this research, we targeted households with pregnant women and or women with children of five years and under, and this was especially exemplified through the key informant technique [35,36]. A limitation of this study was substantial non-compliance of subjects, where about 280 women were visited for interviews but some women were not willing to participate in the surveys; thus, limiting the achievement of the targeted sample size.

The questionnaire included open-ended questions on the dietary practices of the women during pregnancy, especially related to avoidance or consumption of some foods due to cultural beliefs (food taboos), dietary preferences and/or both, and the use of wild foods, additives/medicine or supplements, specifically to enhance maternal and child health. Individual food consumption behaviour was determined from the women’s responses: whether their food consumption practices during pregnancy were governed by their cultural beliefs, dietary preferences or both. Participants were also asked to ascertain whether or not they believed in traditional taboos that certain foods should not be consumed by pregnant women, whether or not they practiced such beliefs and from where such knowledge came from. Women were to list the various food items that are associated with taboos in their communities, and among those who practiced food taboos, a more detailed interaction was done to ascertain the various reasons for avoiding the foods that were mentioned during pregnancy. Additionally, among those who reported avoiding some foods due to dietary preferences, they also provided a list of the foods they avoided, and a more detailed interaction was also done to ascertain the various reasons for avoiding those foods during pregnancy. Women were then classified into three different groups as the drivers of food consumption, which included those who followed cultural beliefs (food taboos) in determining their food consumption; those whose food consumption patterns were governed by dietary preferences; and lastly, those who followed both culture and food preference when choosing what to consume or avoid during pregnancy. Some sections of the questionnaire determined women’s socioeconomic and demographic characteristics, household food acquisition status and individual food consumption behaviour. 

#### 2.2.2. Focus Group Discussions

The study implemented a qualitative study design in the form of focus group discussions (FGDs) which actively made use of group interaction on the issues relevant to a specific topic [37,38]. Nine FGDs were conducted with 7–12 women of mixed age groups, particularly young and old women. Data saturation (determined by the number of FGDs) was reached when there was enough information obtained from the FDGs to replicate the study when the ability to obtain additional new information was attained, and when further coding was no longer feasible, as we kept getting repeat/identical information. There were 94 participants in total and these were purposively recruited by the community leaders, village leaders or ward leaders, who also helped with organizing the venues and times for the FGDs. Participant selection targeted women who had not participated in the household surveys. The composition of the study’s participants was diverse with regard to age, pregnancy status and being a mother. Sex-specific groupings (females only) were done to ensure that males did not hinder the freedom of female participants to express opinions and openly discuss what are largely deemed as women’s issues. Groups were differentiated by the socio-economic attributes selected, because participants with similar characteristics become more comfortable with each other and can participate freely in the discussions. The FGDs were held in comfortable, peaceful and convenient settings and each FGD took between 60 and 90 min. For each FGD, both the principal researcher and an assistant made notes and recorded all the discussions, after consent was provided by the participants. Most participants had informal employment; those who were formally employed were not available at the times when the FGDs were conducted. We managed to obtain information on the beliefs and feelings of individuals in the Kat River Valley and why they acted in the way they did during pregnancy.

Reflexivity was a critical issue in this study prior to each FGD, as we situated ourselves socially and emotionally in relation to our study participants. We also positioned ourselves as “strangers in a strange land” who were studying the unfamiliar subjects; thus, bringing awareness of unconscious bias to the participants. This was advantageous because the researcher was ‘ignorant’ whilst the respondents were in the expert position; thus, bringing about a knowledge-imbuing experience. Therefore, the study was approached from a fresh and different viewpoint, posing new questions that would lead to innovative directions. However, to minimise the challenges that would have existed due to language sensitivity and the researcher lacking relevant identity or direct experience, and not being able to comprehend what would be happening in the communities—we recruited a research assistant who was familiar with the IsiXhosa culture and its norms. 

All questions asked in the FGDs were open-ended, with new questions arising from the responses given, as participants were able to build on each other’s ideas and comments. The FGDs sought responses to the following core questions:

1. In IsiXhosa culture, when a woman is pregnant, are there any specific foods (herbs/plants/animals) that they should eat for the benefit of either the mother or the unborn child?
2. What are the health benefits to the mother and the child in following this?
3. What happens if the mother avoids consuming these foods?
4. Are there any taboo foods or specific foods (herbs/plants/animals) that a pregnant woman should not consume for the benefit of either the mother or the unborn child?
5. Are these cultural beliefs being followed by pregnant women in your communities?
6. What are the consequences on the health of the expecting mother and the child if these beliefs are not followed?
7. Culturally, what do you do to correct this?

### 2.3. Data Analysis

Data were entered and cleaned using Microsoft Excel and descriptive statistics were obtained using Statistica version 12 (StatSoft Inc., Tulsa, OK, USA). Descriptive data are presented as means and standard deviations (SDs) (mean ± SD) and percentages. At the end of each FGD session, the researcher and the assistant compiled their notes into one document; thus, linking accurately, the statements to anonymously-coded individual identifiers in each group. Data from all the FGDs, which were mostly handwritten field notes, were transcribed into Microsoft Word 13, and everything was translated from IsiXhosa to English, with some important Xhosa words given in brackets. Analysis was first done manually, using the principles of systematic text condensation, following Arzoaquoi et al. [20]. This involved four steps: repeated reviewing of the transcript to gain a thorough sense of the overall content in the texts; identifying central meaningful units in the material; condensing the content through a coding of the text; and finally, creating categories that contained the condensed meaning of the main themes in the material. Because manual analysis of the data may introduce personal idiosyncrasies into themes, we later validated themes from the manual analysis by qualitative content analysis (QCA), which is useful for interpreting textual data content by using a systematic classification process that involves coding to identify patterns or themes. Themes were then analysed through coding [39] using NVivo qualitative data analysis software. Similarities between the codes were identified and those that were connected were combined to form primary themes for the discussions. To ensure that the data analysis was a trustworthy representation of the themes from the FGDs rather than the researcher’s biased reflection, the research assistant and the co-authors were constantly consulted to examine the accuracy of the analysis. Most sections of the discussions were quoted verbatim with a few modifications to increase readability.

## 3. Results

### 3.1. Quantitative Assessments

#### 3.1.1. Sample Description

Of all the 280 households with women visited, 224 women agreed to participate in the surveys, representing 80% of the intended sample size. The mean age of these women was 35.8 ± 11.7 years and 35 (16%) were pregnant at the time of our survey. The mean number of children per household was 3.1 ± 0.9 children. About 25% of the women had finished primary school, with 49% having secondary education and only one percent of women had a post-matric certificate or diploma (Table 1). The majority of the households were female-headed (56%) and 58% of the women were relying on a child support grant only (R410 per month) as their form of cash income, whist 21% did not have any form of cash income to support their families. However, about 72% of the household heads had some form of cash income which supplemented the women’s incomes. Household size for the full sample was 7.3 ± 3.7 persons and household mean food expenditure per month was R1034 ± R576 (Table 1). About 52% of household heads received social grants in the form of child support, an old age pension or a disability pension, whilst 20% were either part-time or full-time employees. 

#### 3.1.2. Food Habits in Relation to Cultural Beliefs and Dietary Preference

A total of 37% of women reported that they considered not consuming taboo foods when they were pregnant; thus, following their culture. About 41% of women followed some personal dietary preferences (mostly governed by like or dislikes, taste, smell of food, allergies, doctor’s orders, etc.) when consuming or planning their pregnancy diet, whilst 39% of women reported that they did not have any food restrictions during pregnancy and that they rarely consumed specific foods to enhance pregnancy. Additionally, about 17% of women had large restrictions, as they considered both culture and dietary preference when consuming or planning their pregnancy diet. Table 2 gives the prevalence of the factors that influence food consumption during pregnancy in the Kat River Valley against age, income and education. The average age of women who had no food restriction was 38.6 ± 12.3 years, which was higher than the mean age observed in the other groups.

#### 3.1.3. Food Taboos

**Figure 1:**
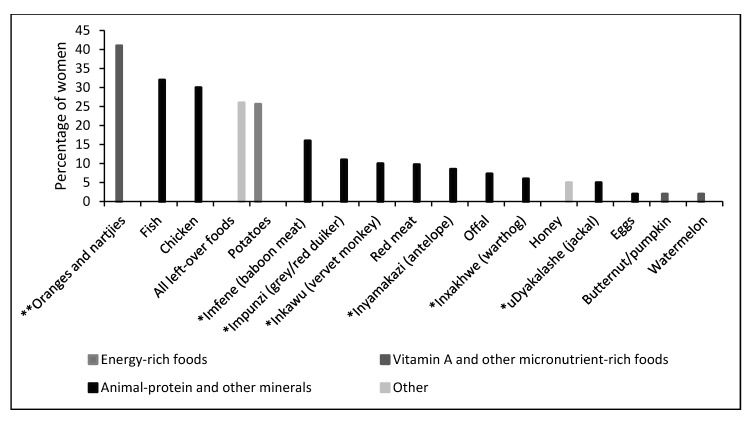
Foods not consumed by pregnant women due to cultural beliefs (food taboos). Wild animals are marked by a single asterisk (*), whilst the double asterisk (**) indicates that this group included orange juices, and other fruits and drinks with an orange colour.

The most commonly-known taboo foods among pregnant women who admitted to following food taboos within the Kat River Valley included oranges, nartjies (*C. unshiu*), orange juices and drinks, chicken, potatoes, fish and wild animals (Figure 1). Foods like beans, eggs, water melon, pumpkin and butternut were cited as taboo foods during pregnancy but were only mentioned by less than 5% of women. 

The reasons for not consuming taboo foods spanned from issues that may affect the mother during delivery, to health issues mostly affecting the child. Others included social problems, including bad behaviour that may affect the child into adulthood. These reasons are detailed in Table 3, together with information on where the knowledge on food taboos was acquired. Knowledge on the taboo foods and the consequences of consuming these foods was primarily acquired from family members, especially grandmothers and mothers. Whilst some women reported that they got the information from the elders in their communities, several women observed a food taboo because of their own experience from previous pregnancies or that of other persons.

#### 3.1.4. Dietary Preference

**Figure 2:**
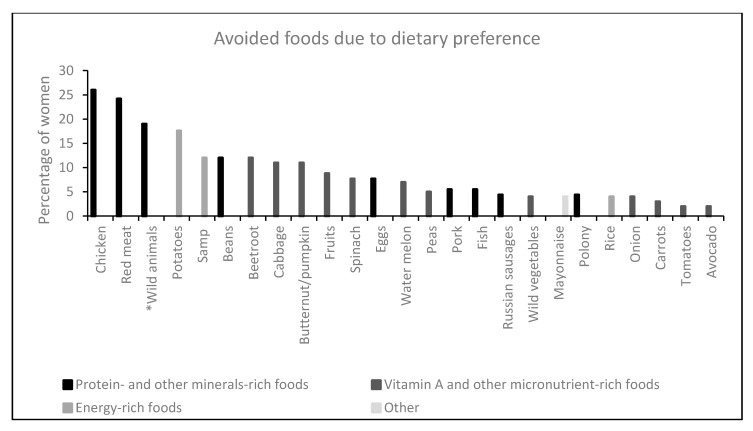
Foods avoided by pregnant women due to dietary preferences. All wild animals were grouped together and these included rabbits, springbok, tortoises, kudu and warthogs.

Whilst 41% of respondents indicated that their food consumption during pregnancy was governed by dietary preferences, most of the foods which they did not prefer eating during pregnancy formed part of the food taboos. For example, about 26% and 24% of women reported that they did not prefer eating chicken and red meat, respectively. Furthermore, wild animals and potatoes, which were also at the top of the list of taboo foods, were reported as not being the favourite foods for some women in the Kat River Valley during pregnancy. All the other foods that the respondents listed as being avoided during pregnancy due to dietary preferences are shown in Figure 2. 

The reasons for not preferring certain foods were explained as being due to the consequences of what the food does to the health of the mother as well as the child. For example, women who avoided chicken and wild animals reported that these foods were not good for the child and the smell made the mothers vomit and always caused morning sickness. Red meat was avoided, as some women perceived that it increased their blood pressure during pregnancy which they deemed as not-good for both the mother’s and child’s health. Reasons like foods causing constipation and heartburn were cited mostly for potatoes, beans and butternut, whilst fish, eggs, pork and onion were avoided by some women due to the “terrible smell” that is produced during cooking. However, some food items were avoided during pregnancy for no specific reasons, as women just did not prefer eating foods like beetroot, cabbage, carrots, avocado, vegetables, fruits and Russian sausages.

### 3.2. Qualitative Assessments: Focus Group Discussions

All participants admitted being knowledgeable about various taboos during pregnancy, including food taboos, and of foods that can be consumed to enhance pregnancy, labour and child health. The common food taboos were reported during each FGD meeting. Participants started by explaining food taboos as the foods that do not go with their culture; foods that they are not supposed to eat as per their customs. Some reiterated that these are governed by the laws introduced by their ancestors not to touch or eat certain food items during pregnancy. However, some participants explained food taboos as those foods which can cause problems or harm to the mother, the baby or even the community if they are consumed. A 24-year-old women from Hertzog explained food taboos as, “*The food that you are not supposed to touch or eat as instituted by our customs,*” whilst an old woman aged 58 years from Balfour said, “*These are the laws that were introduced by our ancestors about the foods that we are not supposed to eat or touch. These foods do not go with our culture to eat or drink and if eaten, can cause harm or problems to the person who ate and may spread to the community.*”

The FGDs revealed all the taboo foods that were mentioned in the surveys and gave the same reasons for being prohibited from eating these foods. These included chicken, baboon, monkey, grey or red duiker, antelope, fish, oranges, potatoes, leftover foods (food from the previous day), offal, honey, beans and eggs. There was also a strong emphasis on eggs and beans as being taboo foods from all the FGDs, especially as perceived by the older generation. A 66-year-old grandmother from Ekuphumuleni community said that, “*Eggs are not supposed to be consumed by pregnant women, as this will cause them to have too much appetite for sex and can search everywhere for it. This may translate to the unborn child if it is a girl.*” Another young woman in her thirties from Hertzog said, “*We are told by our elders that eggs are traditionally taboo for women, especially a just-married wife and pregnant women.*” When women mentioned beans as a taboo food for Xhosa pregnant women, the reasons were mostly related to the baby’s health, although these were different between communities. For example, a 48-year-old mother from Hertzog said, “*Food with beans causes the child to be born with ear problems; therefore, these foods should be avoided,*” whilst a young mother from Blinkwater aged 28 years said, “*If a pregnant woman eats beans during pregnancy, the child’s health will be compromised because beans cause the child to develop sinus infection,*” and another young woman 23 years old and from Ekuphumuleni explained, “*Beans consumed during pregnancy make the baby develop a rash easily*.”

There were also new food taboos that arose from the FGDs which had not been cited in the household surveys. For example, amahewu (a traditional drink made from flour, water and mealie meal) is prohibited during pregnancy in the Xhosa culture, as it is perceived to affect the health of the baby. Amahewu, like potatoes, was perceived to cause breathing difficulties and speech development problems to the child. For example, a 55-year-old grandmother from Herzog explained, “*Drinking amahewu when one is pregnant makes the child develop a blockage on the nose and this causes speech difficulties in the child, who may not say or pronounce some words properly,*” whilst another woman, 44 years old and from Ekuphumuleni, said, “*If you drink amahewu when you are pregnant, the baby is born with breathing difficulties and the speech development of the baby may be affected.*” A young woman in her twenties from Blinkwater indicated that, “*Amahewu causes the baby to have sinus infection,*” and another grandmother, 64 years and from Balfour, mentioned, “*Potatoes and amahewu causes the baby to be born with rash and will constantly suffer from blocked noses which makes breathing difficult for the baby.*”

Fruits, like pineapples, were also revealed as prohibited foods during pregnancy and this was agreed across all locations. A grandmother, 67 years old and from Ntilini, explained, “*Eating pineapple when pregnant may result in the baby being born blind,*” whilst another grandmother from Balfour who was 56 years old said, “*Pineapple causes a child to be born with rash and cracked skin which will be difficult to treat,*” and a young Hertzog mother in her thirties also indicated that, “*Pineapple and honey should not be eaten during pregnancy because if consumed, it causes the baby to develop rash which is difficult to go.*” Peaches were prohibited during pregnancy as explained by a Balfour woman in her forties, who said, “*Peaches affect the baby in the womb and the baby is born with rash or may easily develop rash and ringworm.*” Similarly, guava was also prohibited for pregnant women, as was said by a Blinkwater woman who was 32 years old, “*Guava causes child to develop sores and the child can be born without hair.*”

Aloe juice and comfrey were also mentioned as taboo in the Kat River Valley communities. These are used for stomach cleansing but are regarded as potentially harmful during pregnancy, as these products may kill the foetus or cause premature birth or miscarriage. “*Ukrakrago (aloe) is a cleanser for the tummy but should not be used by pregnant women as this may loosen the stomach and results in premature delivery. Some women use this for abortion,*” said an Ekuphumuleni grandmother. Another grandmother from Ntilini said, “*Aloe juice damages the unborn baby and comfrey also affects the baby. This may lead to miscarriage if consumed by pregnant women.*” Other roots from iphuzi (*Gunnera perpensa* (English name: river pumpkin)), which are used to stimulate vomiting, are also prohibited during pregnancy, as these may induce premature labour. This was also mentioned by a mother from Blinkwater, who said, “*Using roots like iphuzi to stimulate vomiting is prohibited during pregnancy as this may promote miscarriage and one can lose the baby.*”

There were some cultural beliefs other than food taboos that were mentioned during the discussions which pregnant women were prohibited from doing. For example, it was mentioned in all FGDs that a pregnant woman should not walk at night as she may come across the traditional medicines from witchcraft that would result in miscarriage or death at delivery. “*A pregnant woman must not walk at night because she may come across the traditional medicines that would result in miscarriage or death during delivery,*” said Hertzog grandmother. However, pregnant women were encouraged to consume horse womb regularly to protect the mother and baby from witchcraft; and to drink baboon urine, as this is perceived to help the mother with an easy delivery without facing any complications. A mother in her forties from Hertzog said, “*Pregnant women are encouraged to drink baboon urine and eat horse womb from conception until delivery. Horse womb protects mother and baby from witchcraft. The young generation is still using this and it is still working.*” A grandmother in her fifties from Balfour also said, “*Baboon urine and horse womb should be consumed by pregnant women as these help during delivery. Thus, these foods help the delivering mother to deliver well, as everything that should come out will pass easily.*”

As was perceived by the older generation participating in the FGDs, an expectant mother is supposed to drink a solution from a plant locally known as isicakathi (*Salvia scabra* L. f.) from six months of pregnancy until the baby is born. It was perceived that the pregnant mother should put the plant in a container with water and she has to drink half a cup of that water twice a day in the morning and evening. Culturally, that is used as a gauge for the health of the unborn baby, as determined by the manner in which the plant is growing in the container. That is, the healthier the plant is and the better it is growing, the better the health of the foetus, and if the plant dies, then the foetus is expected to die also. A 54-year-old grandmother from Balfour said, “*Pregnant women should drink water from isicakathi every morning and evening from six months of pregnancy until when the baby is born. The plant is kept in a pot with water and the plant growth is monitored. If the plant is growing well, we believe the baby will be growing healthy but if the plant dies, then we accept that the baby will not make it.*” Additionally, once the child is born, they are given isicakathi, which is extracted from putting the plant root in boiling water to make a solution which the baby drinks as herbal tea. “*Isicakathi is given to babies and this helps with wind and phlegm which come out when a baby coughs. Therefore, this medicinal plant helps to clean the chest of the new born so that it will not suffer from these diseases. It also cleans the baby so that it does not suffer from rash and the stomach is cleaned also,*” said a 56-year-old grandmother from Balfour. 

The child is also given inkunzane (*Dicerocaryum senecioides* (common name: Devil’s thorn)), a traditional remedy for many ailments that a child may face due to what could have been consumed by the mother during pregnancy. A 54-year-old grandmother from Blinkwater also mentioned that, “*Isicakathi helps the baby to not be constipated and inkunzane helps with wind, cleanses the stomach of the child and they would not have stomach pains. These medicines help the baby to get rid of all the foods that they eat through the mother during pregnancy.*” Furthermore, the bark of a tree called umthombothi (*Spirostachys africana*) is ground on a coarse stone to form a powder which is mixed with water and is applied on the face of infants to treat the rash which may have developed. The solution is also administered orally in small quantities. The paste also prevents the rash from reoccurring. A 61-year-old grandmother from Hertzog explained, “*Umthombothi, a bark of a tree is used for bathing or drinking to a clear rash that develops on the child. If the rash is not treated, it can cause the child to have blocked nose and ears which causes breathing and hearing problems to the baby.*” Another 62-year-old grandmother from Balfour mentioned that, “*Pregnant women these days no longer eat healthy foods. They eat fish, roasted potatoes and peaches which affect the baby when they are in their mother’s womb. Children are now born with many health problems, such as ring worm and many other diseases because their immune system is not strong. Isicakathi, inkunzane or umthombothi can help ease these diseases.*”

## 4. Discussion

Food taboos, especially among women in sub-Saharan Africa, have been identified as one of the factors contributing to maternal undernutrition during pregnancy [10,12,18,40]. According to the UNICEF Food-Care Health conceptual framework, cultural norms, taboos and beliefs lie within the contextual factors included as one of the basic causes of malnutrition [13,41]. This is because poor nutritional practices, especially during pregnancy and early childhood, can have marked consequences for children’s growth and development. In many communities, pregnant women have to follow some cultural taboos and practices, which influences the food they eat; thus, making the women more vulnerable to several micronutrient deficiencies, especially vitamin A, folate, iodine, iron, calcium and zinc, all of which are crucial during pregnancy [10,42,43]. However, pregnant women require a variety of energy-rich and nutrient-rich foods, such as animal products, fruits and vegetables for the health of both the mother and the developing foetus.

Approximately 37% of respondents in this study reported avoiding one or more foods during pregnancy based on the local food taboos. This is similar to many studies reported elsewhere, where women would adhere to different food taboos and beliefs, with some of the rejected foods being nutritious foods which can interfere with adequate prenatal nutrition [10,12,13,18,40]. Our study revealed that the foods most commonly avoided during pregnancy were meat products (chicken, wild/bush meat and red meat), fish, potatoes, fruits (oranges, nartjies and other fruits with an orange colour, peaches, pineapples and guava), beans, eggs, butternut and pumpkin. Most of the foods that were reported as taboo foods are rich sources of essential micronutrients (beans, eggs, offal, all fruits, pumpkin and butternut), protein (bush meat, fish, eggs, chicken, offal and beans) and carbohydrates (potatoes and amahewu), which are crucial for maternal health and child development. Alarmingly, some of the restricted foods are from most vital food groups, leaving these pregnant women with limited dietary diversity and vulnerable to many nutrient deficiencies, including of micronutrients, which may lead to malnutrition during pregnancy. This has been reported elsewhere in some African [10,12,13,18,40] and Asian [22,24,25] countries, where an entire food group can be restricted during pregnancy. However, the reasons behind avoiding consuming these foods differed with communities. For example, women of Fulla ethnicity in Gambia are prohibited from eating several types of food rich in carbohydrates, animal proteins and micronutrients during pregnancy for various reasons, which is contributing to the high protein/caloric malnutrition during childhood and pregnancy in the country [12]. Poor nourishment during pregnancy has also put many mothers at high risk during delivery [44].

According to the 2018 Global Nutrition Report [5], South Africa is one of the countries with a triple burden of malnutrition. That is, the country has high rates of childhood stunting, anaemia in adult women and overweightness in adult women. The latter is mainly due to women eating too many refined grains; sugary, fatty and salty foods; and not enough foods that promote health, such as fruits, vegetables, legumes and whole grains [4]. About 56% of South Africa’s population live in poverty and almost 28% in extreme poverty (below the food poverty line) [31], which is also the case for most women in the Kat River Valley. Poorer households typically have less diverse diets. This could be exacerbated through the additional burden of food taboos. Considering that 21% of women in the study did not have any source of cash income, 58% depended on child support grants of R480 per month (although the income from the household head would supplement the income), the mean household food expenditure was R1034 and there was an average of seven persons per household, affording sufficient quantities and a quality diet might only be a wish for many women in the Kat River Valley. Factors such as age, education level and socioeconomic status have been noted to influence women’s awareness about the importance of a balanced diet and healthy eating during pregnancy [16,17,40]. However, our study showed no significant differences in education level and income status of women who followed food taboos and those who did not which is contradictory to Oni and Tukur [40] and Vasilevski and Carolan-Olah [17].

Some pregnant women in the Kat River Valley avoided certain foods for different reasons which included pregnancy outcome, the birthing process, avoiding undesirable body forms of the babies and out of respect for their elders if instructed to do so. Some animal-product foods (meat, fish and eggs) and beans, which are good sources protein, vitamins and minerals were avoided for the fear of having a disabled child, a baby behaving like the animals, a child becoming a thief and the fear of early maturity for the child. These foods were also avoided to protect a child from being born susceptible to many diseases, including respiratory problems, eczema, ear problems, boils, rashes, wounds, falling out/no hair, always losing nails and the baby having problems with the umbilical cord. Potatoes, amahewu and honey, which are good sources of calories, are not consumed because of the fear of having a baby with breathing and sinus difficulties, compromised speech development and bad skin due to rashes. Furthermore, the other food taboos restricted the consumption of fruits which are highly nutrient-dense, especially providing vitamins A and C. Fruits which are not eaten during pregnancy included oranges, nartjies and all other fruits with an orange colour for the fear of birthing a baby with skin and eye discolouration. Fruits such as pineapples, peaches and guavas are not consumed during pregnancy because it is believed that the baby will be born blind; may have cracked skin; or may develop ringworm, sores and rashes, which can be difficult to cure during childhood; and that the baby will be born bald.

Other studies have also reported the avoidance of some of these foods during pregnancy. For example, Zepro [16] reported the avoidance of honey, oranges and pineapples in Ethiopia for reasons linked to having a baby with discoloured skin; inducement of abortion or stillbirth; and to protect the baby from contracting worms, malaria and diarrhoea during childhood. Cherkos et al. [15] reported on the avoidance of potatoes by pregnant women to avoid having big babies, who can cause labour difficulties. Fish is also a taboo food during pregnancy in Tanzania [23], in Indonesia [24] and in Malaysia [25], as it is believed to cause difficulties during delivery. Contrary to these studies, some women in the Kat River Valley believed that difficulties during delivery are caused by consuming leftover foods or walking at night during pregnancy. Consumption of meat, vegetables and chicken eggs during pregnancy is also taboo in Indonesia [24] and the Temiar women in Malaysia believe that consuming antelope meat may cause convulsions and prolonged labour [25]. Consumption of eggs is prohibited in Nigeria because it is feared the children may develop bad habits after birth [13,18], which is different from the IsiXhosa culture, where eggs are believed to increase the mother’s appetite for sex, which can then be transferred to the unborn female child. Therefore, due to all these restrictions, pregnant women are exposed to a diet that is reduced in essential nutrients. The amount and quality of food the pregnant woman can choose and consume is limited; thus, potentially reducing the dietary diversity, to the potential detriment of both the mother and the unborn child.

The knowledge of the food taboos and the negative consequences that may occur if not obeyed, especially during delivery and birth outcomes, has been passed on for generations in the Kat River Valley. The majority of women have reported acquiring this knowledge from their grandmothers, elderly people in their communities and their mothers, although few individuals reported having experienced these consequences. Sharifah Zahhura et al. [25] and Mohamad and Ling [26] reported on how knowledge of food taboos and the consequences of consuming these foods have been passed from generation to generation. As perceived by women in the study, most of these food taboos are based on learned behaviour, which is acquired mostly by instruction from grandparents, parents, family members or observation from close relatives and friends who practice it.

This study has shown substantial overlap between non-preferred foods and taboo foods, as most of the foods mentioned as non-preferred were also listed as taboo foods. For example, chicken, red meat, wild animals, potatoes, beans, butternut/pumpkin, fruits, fish and eggs, which were the top taboo foods, also appeared as the top foods that the majority of women did not like to consume during pregnancy. Thus, cultural beliefs and food taboos within the Kat River Valley had a strong influence on the choice of food and food consumption behaviour of many women during pregnancy. For example, most women in the Kat River Valley who identified themselves as “not followers of culture” preferred not to consume the foods which were identified as taboo foods in their communities. Although the reasons for avoiding these foods were different from those cited for avoiding taboo foods, some women avoided consuming the “not preferred” foods for no specific reasons. Like in other African countries, many pregnant women in the Kat River Valley abstain from some nutritious foods as part of their traditional food beliefs, echoing the study by Patil et al. [45] and some unintentionally follow food taboos which significantly contributed to their food choices through preferences.

There have not been any empirically established relationships between the consumption of the foods listed as taboo foods and the aforementioned consequences. Some women perceived themselves to have had experienced the consequences for not obeying the traditional beliefs. They believed that the many health problems, such as ringworm and the compromised immune systems of the young children in their communities were linked to the consumption of some of the taboo foods, such as fish, potatoes and peaches by the expectant mothers. Additionally, the consumption of aloe juice has been mentioned as a taboo that protects both the mother and unborn child, as this may lead to abortion or premature delivery if consumed during pregnancy. Henrich and Henrich [46] also showed how food taboos for pregnant and lactating women in Fiji selectively targeted the most toxic marine species; thus, effectively protecting both mother and child from fish poisoning. Furthermore, medicinal plants were shown to play a significant role during pregnancy, birth and postpartum care in the study. The use of plants to ensure good development of pregnancy and facilitate labour is a particularly well-established practice in Africa [47], with some women consuming these plants to strengthen pregnancy, promote foetal growth and make delivery easier [29]. Some women in the Kat River Valley also make use of plants to make herbal decoctions as traditional remedies for many ailments, strengthening pregnancy and for overall health of both the foetus and mother. The consumption of horse womb and drinking baboon urine for strengthening pregnancy, avoiding miscarriage or immature delivery and promoting safe delivery was also mentioned in the study. However, there is no known scientific basis to substantiate this belief and while some women claimed to drink baboon urine, this seems an unlikely scenario in reality. For example, questions would arise on how these women would get a regular supply of urine from a wild baboon for the entire pregnancy period; thus, seemingly a rather far-fetched tale when one thinks about it.

This study has both strengths and limitations. An important strength is the use of a mixed methodology with a good representative sample to investigate the drivers of food choices and dietary preferences in the Kat River Valley. This makes it possible to replicate the study with other IsiXhosa people in other regions, and with other different cultures within South Africa; thus, allowing a direct comparison within and between cultures. Another important strength of this study was providing stronger evidence for a conclusion through convergence and corroboration of findings from both qualitative and quantitative research which added insights and improved understanding that would have been missed when only using a single method. For example, some of the information on food taboos like amahewu, pineapple, guava and the use of other herbs and the perceived consequences which had not been mentioned during the household surveys came out during FGDs. This could, however, increase the generalizability of our study results because we assumed a more complete knowledge was necessary to inform theory and practice. An important limitation of our study was conducting the study in five small villages under one district/municipality in the Eastern Cape province, which may not be representative of the entire province and/country. Cultural beliefs may also have had an impact on our results, as some women were conservative and reluctant to talk about their food beliefs and dietary practices during pregnancy, as this was regarded as exposing their culture, especially to someone regarded as a stranger. This may have led to an underestimation of women’s participation in cultural practices and the use of medicinal plants/herbs during pregnancy. Therefore, our results need to be interpreted with those advantages and limitations in mind.

## 5. Conclusions

Pregnant women in developing countries are considered to be nutritionally vulnerable, as they are often subjected to different degrees of nutritional stress, and those who follow traditional food taboos have increased chances of developing a range of negative pregnancy outcomes, including compromised health of the baby in future. This can be reduced through encouraging pregnant women to consume diverse and healthy diets with essential micronutrients. For example, adopting a diverse, healthier and more sustainable diet with emphasis on locally produced fruits, vegetables and protein rich foods (legumes, seeds and nuts), and with the inclusion of limited amounts of foods of animal origin, may significantly improve maternal nutrition and the health of the baby. Therefore, there is a role for nutritional education for women in the Kat River Valley because this should significantly increase the nutritional knowledge of pregnant women who receive it. Adhering to culturally-appropriate nutrition education may be an important care practice for many pregnant women in the Kat River Valley, who could be vulnerable to poor nutrition. However, understanding the food taboos among the isiXhosa people helps in designing appropriate nutrition intervention programmes for the Kat River Valley communities which target maternal and child malnutrition in a cultural context.

## Figures and Tables

**Table 1:** Sample distribution by demographic characteristics.

	Indicator	Category	% (*n* = 224)
Maternal characteristics	Location in Kat River Valley	Hertzog	20
		Balfour	35
		Ekuphumuleni	6
		Blinkwater	25
		Ntilini	14
	Women’s mean age (years)	35.8 ± 11.7	
	Maternal education	Primary (up to grade 7)	25
		Secondary (grade 8–11)	49
		Matric (grade 12)	25
		Post matric	1
	Maternal source of income	None	21
		Child support grant only	58
		Child support grant and other sources	5
		Government grant	8
		Pension	8
Household characteristics	Household head	Female-headed households	56
		Male-headed households	44
	Household head source of income	None	28
		Old age grant	7
		Child support grant	6
		Pension/Government grant	39
		Part-time/full time job	20
	Mean household food expenditure per month	R1034 ± R576	
	Mean household size	7.3 ± 3.7 persons	

**Table 2:** Factors influencing food choice and consumption by pregnant women in Kat River Valley, as per women’s socio-economic characteristics.

Drivers of Food Choice and Consumption	* Number of Women (*n* = 224)							
	No Food Restrictions		Culture		Dietary Preference		Culture + Preference	
	N 88	% 39	N 82	% 37	N 91	% 41	N 37	% 17
Women’s mean age (years)	38.6 ± 12.3		34.5 ± 10.8		33.1 ± 10.7		33.1 ± 9.5	
**Educational Status (% of Women)**								
Primary (up to grade 7)	13		7		9		4	
Secondary (grade 8–11)	17		20		22		10	
Matric (grade 12)	9		10		10		3	
Post matric	-		-		-		-	
**Source of Income (% of Women)**								
None	9		5		9		3	
Child support grant only	21		24		24		10	
Child support grant + other sources	1		4		4		3	
Government grant	5		2		1		-	
Pension	4		2		3		2	

* More than one answer to this question was given. Percentages were taken out of *n*.

**Table 3:** Most-cited reasons for considering certain foods as taboo foods and where the knowledge was acquired.

Taboo Foods	Reasons Why Foods Should Not Be Consumed	Source of Knowledge
Oranges, nartjies and orange juices.	-The baby will be born with yellow skin and will have yellow pimples/rash as well as yellow eyes with cracked lips which is not normal.	-Grandmother
Fish	-Fish makes the baby unhealthy as the baby is born with scales and rash on skin. -The baby will suffer from eczema and may be born with no hair which might fail to grow. -The baby may have rough skin with small pimples.	-Mother and grandmother -Mother’s own experience -Old people in their community
Chicken, including chicken heads and feet	-Baby would not want to sleep or may sleep for less than necessary -The child may be restless and may grow up loving to walk a lot like a chicken which may translate into adulthood. -Baby may not grow hair, will not listen and will always be doing rude things (naughty child).	-Grandmother
Umbeko (any left-over foods)	-Causes delay during labour and the baby may be born with disabilities. -Can delay delivery of the child and mother and child may die. -The mother may pass faeces first during delivery and the baby may be a twin to that which gives bad luck to the child for life.	-Grandmother
Potatoes	-Causes the baby to develop rash -The baby is born with breathing difficulties and may discharge yellow mucus -Affect speech development of the baby and may have difficulties to talk	-Mother’s own experience -Grandmother
Imfene (baboon meat)	-Child will be arrogant to mother and elders -Makes baby to be harsh or naughty and causes the child to steal -Child would be born with stubbornness traits and being naughty.	-Mother’s own experience -Grandmother
Impunzi (grey/red duiker meat)	-Causes ishimcca to the child, i.e., when one always loses the nails. -The child would develop sores or boils on the head -The baby may develop wounds around the stomach and would not have hair	-Mother -Grandmother
Inkawu (vervet monkey meat)	-Makes the baby/child to be naughty -Child would not listen and behave like a monkey -The baby is born disabled	-Grandmother
Red meat	-Makes the mother ill	-Old people in the community
Inyamakazi (antelope meat)	-It makes the baby to have a rash on skin and may not have hair -The baby will do everything that animal does; behaves like an antelope	-Grandmother -Own experience
Offal	-The baby’s umbilical cord will be large and will not fall easily	-Grandmother
Inxakhwe (warthog meat)	-Makes the child to grow up being rude	-Grandmother
Honey	-Causes the baby to develop rash -Baby develops respiratory problems and may have phlegm	-Grandmother
uDyakalashe (jackal meat)	-Makes the child to be naughty and to steal	-Grandmother
